# Supplementary material for: Taxonomy and conservation of grassland earless dragons: new species and an assessment of the first possible extinction of a reptile on mainland Australia
Source: R Soc Open Sci. 2019 May 22;6(5):190233. doi: 10.1098/rsos.190233 (PMC6549961; doi:10.1098/rsos.190233)
Supplement: NOMENCLATURAL APPENDIX [file rsos190233supp1.docx]

NOMENCLATURAL APPENDIX S1

*Tympanocryptis lineata* was originally described based on three syntypes, a situation that requires selection of one as the lectotype to permanently fix the name to one specimen. As part of their wide-ranging revision of the entire Australian herpetofauna, Wells and Wellington (1985) chose a lectotype for *T. lineata*. In doing so they introduced unforeseen nomenclatural issues. The International Code of Zoological Nomenclature current at the time of Wells and Wellington’s publication (International Commission of Zoological Nomenclature 1964, 1985) recommends, but does not mandate, the data set that should be provided with the designation of a lectotype (Article 74, Recommendations A-F). Wells and Wellington (1985) did not follow any of these recommendation; they made no statements regarding the origin of the specimens (available in the type description, which was evidently not read), made no attempt to ensure that the lectotype designated would contribute to stability of the name as widely used in the literature, provided no evidence that they had examined any of the syntypes, nor obtained any morphological data on any of them. Instead they appear to have been guided by the brief entry in Cogger et al. (1983) regarding the identity of the three syntypes of *T. lineata* in the Zoological Museum of Berlin, ZMB 740, and ZMB 4714 (originally two specimens, one of which is now re-numbered 54549), and simply selected one of them, ZMB 740, sight unseen, to be the lectotype. Cogger et al. (1983) listed the type locality as Buchsfelde (sic), the home of the collector, Richard Schomburgk who had arrived in Adelaide in 1849 (Middelmann, 1976), and it is possible to read this entry and assume, incorrectly, that the three specimens were from that locality. Buchfelde, renamed as Loos during the First World War (Manning, 1986), is 4.5 km W of the major South Australian town of Gawler. It is this region of South Australia that has always been considered the type location for *T. lineata,* with its distribution extending from western Victoria, western NSW and much of South Australia.

Data associated with the Berlin *T. lineata* specimens confirms that ZMB 4714 and 54549 were both received from Schomburgk, but ZMB 740 was collected by the explorer John Lhotsky (Lhotzki in German records). This accords with Peters’ (1863) original description which he based on the two specimens received from Schomburgk plus another from the “Lotzkischen Sammlung” (Peters, 1863, p. 230). Unfortunately the only collecting locality information for ZMB 740 is New Holland and S. Australia (P.A. Rawlinson notes), “S. Australia” being short for southern Australia, not the state of South Australia, which was not established until two years after Lhotsky’s specimen was collected. Lhotsky was based initially in Sydney from 1832, made important exploration expeditions in 1834 through the Australian Alps and the adjacent high country of south-eastern New South Wales and eastern Victoria (Wakefield 1975), then moved to Tasmania in October 1836 where he struggled with debt, and finally left permanently for Britain in April, 1838 (Whitley 1967, G.M. Shea, pers.com.). During his time in Australia, the only places Lhotsky was documented to have been in a position to encounter *Tympanocryptis* are the south-eastern highlands, south and west of Sydney, New South Wales; there is no record of him ever travelling as far west as South Australia, which was only settled for the first time in December 1836. ZMB 740 cannot have been collected at Buchfelde/Loos, South Australia, but instead must have come from one of the populations currently associated with the southeastern Australian Grassland Earless Dragons. According to Lhotsky’s journal account of his journey into the Australian Alps he spent a number of days on the Limestone Plains (now Canberra) and also the Monaro Plains, exploring and collecting. Although he provides no account of lizards, Lichtenstein (1837, p.6), who catalogued Lhotsky’s collection for the museum in Berlin, described two lizards in alcohol – a *Scincus* n. sp. and a *Lacerta muricata* – both given the catalogue number 28. Although misidentified as a Jacky Lizard (*A. muricatus*), the catalogue number 28 given to this agamid lizard is that now recorded in the Berlin register for ZMB 740 (F. Tillack, pers. com.). The available documentation and historical evidence thus leave little doubt that ZMB 740, the lectotype for *T. lineata*, was collected by Lhotsky on his collecting trip into the Australian Alps.

References

Cogger HG, Cameron EE, Cogger HM. 1983 *Zoological Catalogue of Australia. Volume 1. Amphibia and Reptilia.* Bureau of Fauna and Flora, Canberra.

International Commission on Zoological Nomenclature. 2012 *International Code of Zoological Nomenclature* *online*. Fourth Edition. Available at: <http://www.nhm.ac.uk/hosted-sites/iczn/code/> (Previous editions 1964, 1985, International Trust for Zoological Nomenclature, London).

Manning G. 1986. *The Romance of Place Names of South Australia*. Privately published, Adelaide.

Middelmann RF. 1976. Schomburgk, Moritz Richard (1811–1891). *Australian Dictionary of Biography* **6**, 91-92.

Peters W. 1863 *Monatsberichte der Königlichen Preussischen Akademie der Wissenschaften zu Berlin* **1863**, 228-236 [1864 on title page].

Wakefield NA. 1975 Dr. John Lhotsky's two excursions into the Australian Alps. *Victorian Naturalist* **92**, 228-243.

Wells RW, Wellington CR. 1985 A classification of the Amphibia and Reptilia of Australia. *Australian Journal of Herpetol*ogy (Supplement Series) **1**, 1–61.

Whitley GP. 1967 Lhotsky, John (1795-1866). *Australian Dictionary of Biography* **2**, 114-115.
