# Supplementary material for: Taxonomy and conservation of grassland earless dragons: new species and an assessment of the first possible extinction of a reptile on mainland Australia
Source: R Soc Open Sci. 2019 May 22;6(5):190233. doi: 10.1098/rsos.190233 (PMC6549961; doi:10.1098/rsos.190233)
Supplement: Sighting data used for T. pinguicolla in sEXTINCT analysis [file rsos190233supp3.docx]

APPENDIX S3

**Table S3.** Sighting data used for *T. pinguicolla* in sEXTINCT analysis. Confirmed records based on museum vouchers (those with confirmed year of collection included in analyses) and additional observational data from Victorian Biodiversity Atlas (VBA). Museum acronyms: Museums Victoria (NMV) and South Australian Museum (SAMA). We used two datasets in analyses: (a) a dataset of confirmed sightings based on museum voucher specimens and sightings with high confidence based on advice from researchers conducting survey work on *T. pinguicolla*; and (b) a data set including all records from the Victorian Biodiversity Atlas.

| Institution | Museum Registration # | Year | Locality | Latitude | Longitude |
| --- | --- | --- | --- | --- | --- |
| NMV | D1847 | ? | Melbourne, Essendon | -37.77 | 144.9 |
| NMV | D1848 | 1885 | Melbourne, Essendon | -37.77 | 144.9 |
| NMV | D1849 | 1885 | Melbourne, Essendon | -37.77 | 144.9 |
| NMV | D724 | 1884 | Melbourne, Essendon | -37.75 | 144.9 |
| NMV | D1338 | ? | Melbourne, Essendon | -37.77 | 144.9 |
| NMV | D1339 | ? | Melbourne, Essendon | -37.77 | 144.9 |
| NMV | D14615 | 1960 | Melbourne, between Rockbank & Werribee | -37.73 | 144.65 |
| NMV | D1506 | 1908 | Melbourne, Yarra River, Good Island (Coode Island?) | -37.82 | 144.97 |
| NMV | D15299 | 1967 | Little River, You Yangs | -37.95 | 144.5 |
| NMV | D3482 | 1906 | Melbourne, mouth of the Yarra River | -37.83 | 144.92 |
| NMV | D3483 | 1906 | Melbourne, mouth of the Yarra River | -37.83 | 144.92 |
| NMV | D5391 | 1884 | Melbourne, Moonee Ponds | -37.77 | 144.92 |
| NMV | D5426 | 1872 | Melbourne, Prahran | -37.85 | 144.98 |
| NMV | D7702 | 1885 | Melbourne, Essendon | -37.77 | 144.9 |
| NMV | D994 | 1912 | Port Melbourne | -37.83 | 144.93 |
| SAMA | R2468.A | ? | Southern Victoria | - | - |
| SAMA | R2468.B | ? | Southern Victoria | - | - |
| SAMA | R2468.C | ? | Southern Victoria | - | - |
| Published account^1^ |  | 1968 | Newcomb, Victoria | - | - |
| Published account^1^ |  | 1969 | Thompson Rd, North Geelong, | - | - |
|  | Observation # |  |  |  |  |
| VBA | 1004467 | 1990 | ROUGHLY 2 KM S OF KIRK BRIDGE | -37.9311 | 144.4793 |
| VBA | 1004481 | 1990 | KIRK BRIDGE | -37.9311 | 144.4781 |
| VBA | 1004271 | 1990 | KIRK BRIDGE | -37.9283 | 144.476 |
| VBA | 1004477 | 1990 | ROUGHLY 2 KM NW OF REDSTONE HILL | -37.6147 | 144.7415 |
| VBA | 1002911 | 1988 | ROUGHLY 2 KM NE OF DONNYBROOK | -37.5094 | 144.9981 |

^1^Pescott T. 1969 Earless Dragon Lizard. *Geelong Naturalist* **6**, 57.
